# The Medical Profession—Quackery

**Published:** 1846-10

**Authors:** 


*The Medical Profession—Quackery.*—The monster evil, of a social kind, which affects the medical profession, is the general poverty of its members; no further proof of this need be adduced than the immense number of qualified assistants, who are everywhere ready to take service at less salary than that obtained by ordinary clerks, or even the superior classes of servants. The numbers of the profession, relative to the general population, is frequently complained of, and none can deny that the legally constituted bodies have most reprehensively suffered pretenders to medical knowledge to spring up on all sides of the regularly educated practitioners of every grade, many of them armed with degrees which may be had for mere purchase, and which are in no wise a test of medical proficiency. But setting aside, in a great measure, this source of mischief, we believe that the legitimate funds which should of right be contributed by the nation to the medical faculty would be amply sufficient to provide for every respectable individual in this country, having any title, legal or otherwise, to call himself physician, surgeon, or apothecary.

The profession is robbed, not by thousands, or by tens of thousands, but by hundreds of thousands sterling, annually. This may seem startling at the first blush, but let us consider the modes in which the funds, which should enrich the profession, flow into illegitimate channels; consider how the public, the other professions, and the government, trench upon its rights, and it will no longer be matter of doubt. Take the sums paid annually to knavish pretenders, who are impudently engaged in practice without the slightest show of right; add to this the sums taken by prescribing druggists; add again the yearly sums paid by the public for patent medicines; to these add the immense sums by which the profession is underpaid by the government for services rendered to the state, or by public bodies, as in the case of the New Poor-Law Commission;—take these, and numerous other items, into consideration, and it will be seen that we have not dealt in figures of speech, but in plain figures of arithmetic.

The obvious mode of relieving the profession of that poverty which these and other causes have rendered so prevalent, is to raise the qualification for practice by raising the standard of medical education, and rendering it penal for unqualified persons to engage in practice. Then, this good effected, the boundaries of the profession must be enlarged, by a close examination of all those points in which the evident functions of the medical faculty are performed by other persons; and this scrutiny must be followed by a determination to assert the rights of the profession. To show our meaning, we need only refer to a few points which, together with others, we shall have hereafter to consider seriatim. There is the quarantine system; the office of coroner; and the entire range of medical statistics: the regulation of lunatics and lunatic asylums; sanatary commissions of various kinds:—all these departments of the public service ought, if the public welfare were alone considered, to belong to the domain of the medical faculty. These things belong to us of right and justice, and can and may be had for the asking. The profession is like a big boy at school who has been set upon by his fellows, but who has only to put out his strength to take at once his proper position.

The monster cause of poverty and degredation is quackery; and this we confidently believe could not exist in its present rampant state but for the countenance afforded to it by members of the profession. Often, indeed, this is done, without improper motive,—unsuspectingly, in fact, though the bane is not the less. Low views have crept in, and the very highest members of the profession often give direct and indirect aid to empiricism, without dreaming what they do. They will recoil from the consequences of their own act when placed nakedly before them. It will be a grand object to separate legitimate medicine from quackery. When this can be done, quackery by itself is such a monstrous bubble that it must shrivel of itself to nothing. But until this is accomplished, legislation will be in vain, and quackery will continue respectable. We shall begin the task by putting our finger upon every glaring or covert professional of empiricism open to observation, not, meanwhile, sparing the unprincipled public quacks themselves. If this separation were effected, and quackery stood bare to public scorn, the profession might then ask for the abolition of patent medicines; till this is the case, the proposal will only be met by ridicule.

Time will show whether the great objects we have proposed are attainable in the present day; whether the professional boundary may be so enlarged as to include a vast amount of emolument and honor now absorbed by extra-professional persons, and whether the immense funds which are at present diverted into improper channels may not be regained for the profession.

Professional opinion, and the action born of strong opinion, are the powerful levers which must be moved to effect these things. It may be a difficult task, but in our labors we shall be animated with a bold hope for the issue.—*Lancet.*—*Med. News.*

